# Allergic Fungal rhino-sinusitis frequency in chronic rhino-sinusitis patients and accuracy of fungal culture in its diagnosis

**DOI:** 10.12669/pjms.36.3.1661

**Published:** 2020

**Authors:** Nukhbut-Ullah Awan, Khalid Muneer Cheema, Fatima Naumeri, Samina Qamar

**Affiliations:** 1 Dr. Nukhbut-Ullah Awan, FCPS. Department of ENT (Unit-II), King Edward Medical University/Mayo Hospital, Lahore, Pakistan; 2 Professor Dr. Khalid Muneer Cheema, FCPS, DLO. Department of ENT (Unit-II), King Edward Medical University/Mayo Hospital, Lahore, Pakistan; 3 Dr. Fatima Naumeri, MCPS, FCPS. Department of Pediatric Surgery, King Edward Medical University/Mayo Hospital, Lahore, Pakistan; 4 Dr. Samina Qamar, FCPS. Department of Pathology, King Edward Medical University/Mayo Hospital, Lahore, Pakistan

**Keywords:** Allergic fungal rhino-sinusitis, fungal culture, Bent and Kuhn diagnostic criteria, chronic rhino-sinusitis

## Abstract

**Objective:**

To determine the frequency of Allergic Fungal Rhino-sinusitis (AFRS) in Chronic Rhino-sinusitis (CRS) patients and the accuracy of fungal culture in diagnosing AFRS.

**Methods:**

Immunocompetent patients with CRS and without invasive fungal rhino-sinusitis presenting over a period of 3 years in ENT department of Mayo Hospital, from April 2014 to September 2017 were included in the study. AFRS was diagnosed clinically and on Bent and Kuhn diagnostic criteria. All patients underwent endoscopic sinus surgery. Removed tissue histopathology and fungal culture was done. Diagnostic accuracy of fungal culture in AFRS patients was determined.

**Results:**

Out of 216 patients of CRS, 45 (20.8%) had AFRS. Mean age of patients diagnosed with AFRS was 29.49±9.16. Out of 45 patients, 26 were male and 19 were female. Nasal polyps were present in 45 (100%) patients, fungal stain was positive in 39(86.7%). CT scan showed sinus expansion in 28(62.2%) patients, heterogeneous opacity in 45(100%) patients and bone destruction in 13(28.9%). Presence of Allergic Mucin was seen in 45(100%) patients, high IgE levels in 36(80.0%), eosinophilia in 21(46.7%), presence of Charcot Leyden crystals in 27(60.0%). Asymmetrical involvement of sinuses was seen in 30 (66.7%) and co-existent asthma was seen in 18(40.0%). Fungal culture positive patients were 25(55.6%). Diagnostic accuracy of fungal culture was 91.6%.

**Conclusion:**

Fungal culture has a key role in confirming diagnosis of AFRS. We also noted that frequency of AFRS is increasing in CRS patients.

## INTRODUCTION

Fungal rhino-sinusitis is a type of chronic rhino-sinusitis (CRS) and has five clinico-pathological variants. Three of these are tissue invasive and include acute necrotizing (fulminant), chronic invasive and granulomatous invasive (indolent) variant. The non-invasive variants include sinus mycetoma (fungal ball) and Allergic fungal rhino-sinusitis (AFRS).^1^ CRS has a prevalence of 2%-16%^2^ and initial reported incidence of AFRS in patients of CRS was 5% to 10% in literature,^3^ however, an increasing incidence in temperate regions have been reported.^4^

AFRS is considered to be a hypersensitivity reaction to fungal antigens.^5^ Gold standard for diagnosis of AFRS in CRS patients is debatable as different forms of CRS may have similar clinical presentation and radiological findings. Most commonly used criteria for diagnosis of AFRS is Bent and Kuhn.^6^

It is a common problem to differentiate actual fungal infection from colonization and histopathology is needed to determine tissue invasion and host reaction to fungus (inflammation, necrosis, hemorrhage), yet on histopathology alone it is difficult to differentiate fungus from allergic mucin. It is thus, recommended to do fungal stain and culture in addition to histopathology for diagnosis of AFRS. Inability to demonstrate fungal organisms in surgical specimens is a major factor to report it as a disease other than AFRS, though the possibility of inadequate methods to culture fungus is mostly ignored. Surgical specimen collection and handling in an optimal way can increase the yield of fungus in removed specimens and this factor should always be considered per-operatively.^7^,^8^

The objective of our study was to determine frequency of AFRS in CRS patients in our set up and accuracy of fungal culture in diagnosis of AFRS. Despite adequate research across the world, there is yet no local data to see increase in frequency and incidence of AFRS and to prove diagnostic importance of positive fungal culture in these patients, so we addressed this gap in our study.

## METHODS

This prospective study was conducted in ENT Department, Mayo Hospital, Lahore from April 2014 to September 2017. The study was approved by the Institutional Review Board (IRB) (909/RC/KEMU, Dated: 05-07-2019) of the King Edward Medical University. Immunocompetent patients diagnosed with chronic rhino-sinusitis with or without nasal polyposis were included in the study after informed consent; while patients with invasive fungal rhino-sinusitis were excluded.

Clinical workup was performed on outpatient basis. Patients’ demographic characteristics were noted. Parameters noted on clinical examination were nasal polyps, laterality of disease and presence of allergic mucin. Raised serum IgE levels (levels more than 300 U/ml) were noted. CT scan of patients was performed and parameters such as heterogeneous opacities, bony destruction, and sinus expansion were noted. All patients fulfilling the major criteria of Bent and Kuhn (any four variables as seen in Table-I), positive examination findings and a suggestive history were labeled as having AFRS.

**Table-I:** Bent and Kuhn Criteria.

Major	Minor
Type 1 Hypersensitivity	Asthma
Nasal polyposis	Unilateral disease
Characteristic CT findings	Bone erosion
Eosinophilicmucin without invasion	Fungal culture
Positive fungal stain	Charcot-Leyden crystal Serum eosinophilia

After an informed consent, all patients underwent endoscopic surgical management under general anesthesia and were operated in reverse Trendlenberg position with 15 degree head up. Nasal endoscopy of all patients was done. Polypoidal mucosa of nasal cavity and sinuses was removed. Nasal cavity was packed with roll gauze soaked in Bismuth iodine paraffin paste. Endoscopically removed sinus mucosa and debris were equally divided into two halves and sent for histopathology and mycological examination and parameters such as eosinophil clusters (eosinophil count higher than 0.3 cells/HPF), fungal hyphae, Charcot Leyden crystals, fungal culture and allergic mucin were noted. Specimen was stained using haemotoxylin and eosin (H&E) and Gomori methanamine silver.

For quantitative variable like age, mean and standard deviation was calculated. While for qualitative variables like Nasal polyps, fungal stain, Allergic mucin, Charcot Leyden Crystals, eosinophilia and IgE level, frequencies and percentages were calculated. The diagnostic accuracy was calculated for the fungal culture by the formula given below:


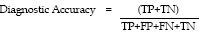


Where TP, TN, FN and FP are as explained in Table-II.

**Table-II:** 2*2 Table for diagnostic accuracy of fungal culture in AFRS patients.

Fungal Culture			

		Positive	Negative
AFRS	Positive	True positive (TP)	False positive (FP)
	Negative	False negative (FN)	True negative (TN)

## RESULTS

Total 216 patients of CRS were included during study period, out of which 45 patients were diagnosed as having AFRS. Frequency of AFRS in patients of CRS was 20.8%. Mean age of patients diagnosed as AFRS was 29.49±9.16, with minimum age 10 years and maximum 44 years. Out of 45 patients, 26 were male and 19 were female with a male to female ratio of 1.3:1. Frequency of nasal polyps, positive fungal stain, sinus expansion on CT scan, heterogeneous opacity on CT scan, bone destruction on CT scan, presence of Allergic mucin, tissue eosinophilia, IgE levels, presence of Charcot Leyden crystals, co-existent asthma and presence of unilateral involvement of sinuses in AFRS diagnosed patients are given in Table-III.

**Table-III:** Different variables’ frequency and percentage in AFRS patients.

Variable	Frequency
Nasal polyps	45(100%)
Presence of Allergic Mucin	45(100%)
Fungal Stain positive patients	39(86.7%)
Heterogeneous opacity on CT scan	45(100%)
High IgE level	36(80.0%)
Charcot Leyden Crystals	27(60.0%)
Eosinophilia	21(46.7%)
Bone destruction on CT scan	13(28.9%)
Sinus expansion on CT scan	28(62.2%)
Positive Fungal culture	25(55.6%)
Co-existent Asthma	18(40.0%)
Asymmetrical involvement of sinuses	30(66.7%)

Diagnostic accuracy of fungal culture was 91.6%.


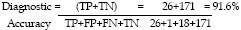


## DISCUSSION

Allergic fungal rhino-sinusitis is a well-recognized disease entity and an increase in its incidence is reported in different parts of the world in last two decades.^9^ History of AFRS is somewhat vague. In 1976, Safirstein was the first who noted combination of nasal polyps with crust formation and a positive sinus culture for Aspergillus.^10^ Robson and colleagues in 1989 introduced the term ‘Allergic Fungal Rhino-sinusitis’ for this disease entity.^11^ Since then, extensive work has been carried out to define the diagnostic criteria for AFRS.

Similarities in clinical presentation of various disease entities like eosinophilic fungal rhinosinusits, eosinophilic mucin rhino-sinusitis and allergic fungal rhino-sinusitis make its diagnosis difficult. Bent and Kuhn criteria is widely accepted criteria for AFRS diagnosis.^12^ We have studied the role of fungal culture in diagnosing patients of AFRS. Similarity in clinical presentation and debatable diagnostic criteria and reportedly increasing incidence of AFRS needs a lot of research for consensus, especially locally.

A significant predictor of AFRS is presentation at a relatively younger age and in our study, mean age of patients was 29.49 years. It is similar to the study of Irshad-ul-Haq et al. where mean age was 31.56 years,^13^ however, the study conducted at Dow Medical College revealed mean age of patients to be 20.75 years indicating much lower age at presentation,^8^ While Jawad et al. reported mean age of patients to be 35.3 years.^14^

Frequency of AFRS in CRS patients in our study was 20.8%. Study conducted by Deutsch E et al in 2004 revealed 4% to 7% patients of AFRS in CRS patients which is significantly lower than our report, indicating that AFRS is increasing in incidence due to specific environmental factors in our part of the globe.^15^ This rising trend has been previously reported in some local and international studies. Study by Rehman AUR et al. in 2009 showed the frequency of AFRS to be 24% which favors our study results.^16^ Another local study in 2015 showed the 22.4% incidence of AFRS.^17^

Geographic factors are believed to influence incidence of AFRS. AFRS cases are mostly reported in regions with high humidity.^18^ The probable explanation of this rising incidence of AFRS in our country is favorable atmosphere for growth of fungal spores and limited health awareness regarding control and complications of allergic rhinitis. Male to female ratio in our study was 1.3:1 and male preponderance in our study is comparable with other local and international literature. 19. Kaur R et al. in their study showed a male to female ratio of 1.18:1.^19^ While a study conducted by Suri N et al.^5^ showed female preponderance which contradicts to our study.

All our AFRS patients presented with nasal obstruction and nasal polyps and this finding is comparable with study of Khattar et al.^20^ In addition to demonstration of fungal elements while diagnosing AFRS, Allergic mucin forms an important diagnostic criterion for AFRS and in present study, Allergic mucin was present in 100%patients diagnosed as AFRS. This finding is comparable with study of Chaitanya et al.^21^ where Allergic mucin was present in 78.26% of patients.

In present study laboratory investigations revealed eosinophilia in 46.7% of patients and IgE levels were raised in 80% of AFRS patients. Eosinophilia and high IgE levels also are considered as important predictors of AFRS and our findings are comparable with many national and international studies.^6^ Radiological investigations play an important role in diagnosing AFRS, although a recent study showed that CT Scan may have some pitfalls in fungal rhino-sinusitis cases, still typical CT findings in patients with AFRS include expansion of involved sinuses, thinning of involved sinus bony walls, heterogeneous opacities and erosion of sinus walls.^22^,^23^ Present study shows heterogeneous opacities in 100%, expansion of sinuses in 62.2% and bone erosion in 28.9% of patients. Presence of fungal elements in removed polypoidal mucosa and debris is another important criterion for diagnosis of AFRS and in our study fungal staining was positive in 86.7% of patients, while fungal culture was positive in 55.65% patients. An international study in 2016 noted 20% patients with positive fungal stain and 91.4% culture positive patients; in contrast to our study.^19^ While study of Montone et al.^24^ showed 74.4% of fungal stain positive patients and 25.5% culture positive patients.

### Limitation of our study

Limitation of our study is that being a hospital based study, we could not calculate the incidence of disease. Also there is a chance of more reported negative fungal culture in our study due to non-viability of fungi in samples, inadequate sample collection from different sites and/or impaction of fungal hyphae in allergic mucin.

## CONCLUSION

Fungal culture has a key role in confirming diagnosis of AFRS. We also noted that frequency of AFRS is increasing in CRS patients.

### Author’s Contribution:

**NA:** Designed, data collection, statistical analysis, final approval of manuscript, and agreement for accountability of all work. **KMC:** Conceived, review of manuscript, agreement for accountability of all work, and final approval of manuscript. **FN:** Did manuscript writing, editing of manuscript, agreement for accountability of all work, and final approval of manuscript. **SQ:** Data collection, statistical analysis, agreement for accountability of all work, and final approval of manuscript.
